# Editorial: Interactions Between Podocytes, Mesangial Cells, and Glomerular Endothelial Cells in Glomerular Diseases

**DOI:** 10.3389/fphys.2022.849693

**Published:** 2022-03-23

**Authors:** John D. Imig, Xueying Zhao, Ahmed A. Elmarakby, Tengis Pavlov

**Affiliations:** ^1^Drug Discovery Center, Medical College of Wisconsin, Milwaukee, WI, United States; ^2^Department of Physiology, Morehouse School of Medicine, Atlanta, GA, United States; ^3^Department of Oral Biology and Diagnostic Sciences, Augusta University, Augusta, GA, United States; ^4^Division of Hypertension and Vascular Research, Henry Ford Health System, Detroit, MI, United States

**Keywords:** glomerulus, podocytes, mesangial cells, glomerular endothelial cells, nephropathy

## Introduction

The complex mechanism by which kidneys fail and progress to end stage renal disease (ESRD) involves renal hemodynamics, glomerular function, and tubular function. A progressive decline in glomerular filtration (GFR) to ESRD ultimately requires dialysis and kidney transplantation (Foley and Collins, 2007). All types of glomerular cells including podocytes that maintain the filtration barrier, mesangial cells that have contractile properties, parietal epithelial cells that serve as podocyte progenitors and glomerular endothelial cells that respond to changes in shear stress and plasma constituents maintain proper glomerular function (Daehn and Duffield, 2021). During renal diseases these glomerular cells and cell interactions become dysfunctional (Daehn and Duffield, 2021). Glomerular diseases are common and include minimal change disease, focal segmental glomerulosclerosis, membranous nephropathy, and lupus nephritis.

This Research Topic captures changes in glomerular cell types resulting in progressive decline in GFR to ESRD. Research publications span cell signaling, animal studies, disease pathology studies, renal hemodynamics, and glomerular function. The Research Topic contains ten contributions that demonstrate the exciting investigations on interactions between podocytes, mesangial cells, and glomerular endothelial cells in glomerular diseases.

## Glomerular Modeling

The complexity of the glomerulus and glomerular filtration barrier has led to modeling that considers the functions of the different cell types. A review article provides an update on the ever-expanding research efforts to model glomerular function (Ebefors et al.). This wave of new modeling technologies includes glomerulus-on-a-chip, three dimensional microfluidic models, and organoids that can enable better predictions of cell-to-cell interactions in the glomerulus. The continuous development of better modeling of the glomerulus is sure to accelerate discoveries that will lead to better therapeutics for glomerular diseases.

## Immunoglobulin a (IgA) and Membranous Nephropathy

Four original research articles to the Research Topic tackle IgA and membranous nephropathy. A major complexity to glomerular diseases is that multiple diseases can occur at the same time in patients. The combination of IgA nephropathy and minimal change were the focus of a retrospective patient cohort study (Li et al.). The findings of this study revealed that low levels of plasma galactose deficient IgA1 (GdlgA1), IgG antiglycan autoantibodies were found in patients with combined IgA nephropathy and minimal change disease (Li et al.). The plasma from these IgA nephropathy and minimal change patients resulted in a weaker inflammatory response when added to mesangial cells than plasma from IgA nephropathy patients (Li et al.). A second study evaluated plasma from IgA nephropathy patients and podocyte injury (Jia et al.). Angiopoietin-like protein 4 (Angptl4) levels in IgA nephropathy patients correlated with podocyte injury (Jia et al.). The next two studies evaluated traditional Chinese medicines on nephropathy. The ability for modified Huangqi decoction (MHCD) to reduce IgA nephropathy in rats was determined (Chang et al.). MHCD reduced proteinuria, decreased mesangial cell hyperplasia and matrix expansion, and increased podocyte number in IgA nephropathy rats (Chang et al.). A study in patients with refractory idiopathic membranous nephropathy demonstrated that Shulifenxiao treatment for up to 2 years had a favorable safety profile, increased the remission rate, and improved glomerular function (Cui et al.). These studies highlight factors that contribute to damaging glomerular cells in nephropathies and potential therapies that target glomerular cells to combat nephropathies.

## Vascular Endothelial Growth Factor Inhibition and Glomerular Toxicity

Vascular endothelial growth factor (VEGF) inhibition on glomerular function is the topic in two articles that are published in this Research Topic. VEGF inhibitors are given systemically to treat cancers and intravitreally for age-related macular degeneration and diabetic retinopathy (Hanna et al.; Jankiewicz et al.). A hypothesis and theory article puts forth evidence that intravitreally administered VEGF inhibitors can cause thrombotic microangiopathy and acute kidney injury (Hanna et al.). This supports the concept that glomerular disease needs to be monitored in patients receiving in intravitreally administered VEGF inhibitors (Hanna et al.). Next, the ability for a dual soluble epoxide hydrolase (sEH) and cyclooxygenase-2 (COX-2) inhibitor, PTUPB to combat hypertension and glomerular injury during systemic administration of the VEGF and multikinase inhibitor sorafenib is demonstrated (Jankiewicz et al.). PTUPB was able to decrease glomerular permeability and improve podocyte and mesangial cell function in rats administered sorafenib (Jankiewicz et al.). Taken together, the glomerular damage induced by VEGF inhibitors needs to be monitored; however, novel therapies to mitigate the glomerular damage are on the horizon.

## Other Glomerular Diseases

Various aspects of kidney and glomerular damage are the focus of a review and two scientific studies published in this Research Topic. Hyperuricaemia which occurs due to alterations in urate production and excretion was the focus of a review article (Sun et al.). Urate transporters in the kidney provide a mechanism for therapies to lower urate levels in hyperuricaemia (Sun et al.). Glomerular nephropathy can be induced by environmental factors falling under the responsibility of occupational safety and health. Trichloroethylene is solvent widely used for degreasing metal, parts cleaning and exposure to this chemical exceeds 20,000 industry and warehouse workers every year in China. Wang and colleagues report signs of renal insufficiency in six patients with occupational medicamentosa-like dermatitis due to TCE. To study mechanisms of renal toxicity of TCE, the authors administer trichloroethylene in Freund's complete adjuvant to Balb/c mice (Wang et al.). The sensitized animals exhibit ultrastructural damage and loss of podocytes, plus increased plasma BUN and creatinine. The involvement of mTOR/cathepsin-L hyperactivity in TCE-induced renal damage is demonstrated by a series of rapamycin treatments and histopathological analyses (Wang et al.). Nephrology patients develop extrarenal abnormalities due to uremia, vitamin D deficiency, electrolyte imbalance and other complications. Hemodialysis brings additional risks—access complications and use of anticoagulants. As a result, dialysis patients exhibit systemic vascular damage and inflammation which erodes the glycocalyx layer. Kusuzawa et al. present a single-center study featuring whether syndican-1, a polysaccharide component of glycocalyx, can be used as a marker of endothelial injury in patients on dialysis. The authors analyze syndican-1 level in relation to the procedure settings, fluid removal, and use of anticoagulants and conclude that assessment of syndican-1 can be used in the selection of treatment and limiting endothelial injury (Kusuzawa et al.).

## Conclusions

The Research Topic *Interactions between podocytes, mesangial cells, and glomerular endothelial cells in glomerular diseases* demonstrates the need for a better understanding of mechanisms that contribute to glomerular function and damage. This collection of ten articles demonstrates that glomerular diseases are diverse and include different glomerular cell types and their interactions. Therefore, there is great need for a better understanding of these glomerular cell types in diseases to allow for the development of effective therapies to combat glomerular diseases.

## Author Contributions

JI conceived the content and drafted the manuscript. JI, XZ, AE, and TP revised and approved the final manuscript. All authors contributed to the article and approved the submitted version.

## Funding

This work was supported by the National Institute of Diabetes and Digestive and Kidney Diseases DK126452 and DK123266.

## Conflict of Interest

The authors declare that the research was conducted in the absence of any commercial or financial relationships that could be construed as a potential conflict of interest.

## Publisher's Note

All claims expressed in this article are solely those of the authors and do not necessarily represent those of their affiliated organizations, or those of the publisher, the editors and the reviewers. Any product that may be evaluated in this article, or claim that may be made by its manufacturer, is not guaranteed or endorsed by the publisher.
